# Milestones and Monitoring

**DOI:** 10.1007/s11899-015-0258-1

**Published:** 2015-04-29

**Authors:** Alessandro Morotti, Carmen Fava, Giuseppe Saglio

**Affiliations:** Division of Hematology and Internal Medicine, Department of Clinical and Biological Sciences of the University of Turin, “San Luigi Gonzaga” University Hospital, 10043 Orbassano, Turin Italy

**Keywords:** CML, BCR-ABL, TK inhibitors, RQ PCR

## Abstract

In chronic myeloid leukemia (CML), the presence of a specific chromosome marker (Ph-chromosome) as well as of the corresponding molecular marker (BCR-ABL fusion transcripts) provides suitable and precise tools to monitor the burden of the disease present at diagnosis and that of the residual disease present at specific time points during treatment. A huge number of studies have clearly demonstrated that in CML cytogenetic and molecular responses are strictly correlated to the final outcome of the patients and the correct use of standardized methods to assess the achievement of specific degrees of disease reduction at specific time points during treatment has become an essential part of proper clinical management of CML. The target to be achieved and the corresponding “optimal response” definition are however evolving, and at least for some patients, they may be represented not only by best possible overall survival (OS) but also by the possibility to discontinue the tyrosine-kinase inhibitor (TKI) treatment and therefore to live in a treatment-free remission (TFR) status. Therefore, at least for some patients, deep degrees of molecular response, as MR^4^ and MR^4.5^, whose precise definition has been recently introduced and that are prerequisites to try to discontinuation, are becoming the target to be achieved even in common clinical practice. As a fast initial decline of the disease burden after therapy start may be highly predictive for the final outcome of patients not only in terms of progression-free survival (PFS) and of PS but also in terms of possibility of achieving deep molecular responses, a more intense and punctual monitoring of the response of CML patients during the first 6 months of TKI therapy is now recommended by the more recent versions of the European Leukemia Net (ELN) and National Comprehensive Cancer Network (NCCN) guidelines, as this represents the major driver to decide therapy.

## Introduction

The degree of leukemia load reduction during therapy is the most important prognostic factor for chronic myeloid leukemia (CML) patients [1, 2].

Various analyses have shown that patients who do not achieve good cytogenetic or molecular responses have a worse outcome, characterized by an increased risk of relapse, of progression, and of death [3, 4]. Based on these principles, a panel of CML experts on behalf of the European Leukemia Net (ELN) as well as members of the National Comprehensive Cancer Network (NCCN) have previously established and continuously revised treatment milestones to be achieved during CML treatment with tyrosine-kinase inhibitors (TKIs) [5••, 6••]. Indeed, criteria based on the degree of hematologic, cytogenetic, and molecular response expected at defined time points (considering that CML responses follow a sequential order that begins with hematologic remission and continues with cytogenetic and molecular response) have been derived from data of different studies showing that patients who do not present a certain degree of response at a given time show a worse progression-free survival (PFS) and overall survival (OS) than those who do it [7•, 8, 9]. Optimal response is intended when the response obtained is the one associated with an optimal OS, and there is therefore no need, in presence of a good tolerance, to change the TKI therapy [5••]. Failure is intended when the residual probabilities of achieving an optimal response are indeed very scarce and therefore, when and if possible, while warning or suboptimal response correspond to intermediate situations between optimal response and failure, in which the reduction of the Ph-positive clone is slower than expected for an optimal response, but there are still substantial possibilities for the patient to achieve the planned degree of response later on [5••]. This obviously implies that an appropriate and timely follow-up with cytogenetic and standardized molecular methods of adequate reliability is essential even in usual clinical practice [10–12]. In particular, molecular monitoring of BCR-ABL transcript levels by real-time quantitative PCR (RQ PCR) is progressively becoming the most precise way to monitor CML patients. With respect to conventional cytogenetic analysis, RQ PCR cannot only allow to monitor the first steps of reduction of the leukemic burden occurring within the first months of TKI therapy, but it may also allow to estimate the amount of the residual disease once complete cytogenetic response (CCyR) is achieved, as the sensitivity that can be reached with the present RQ PCR procedures in a sample of good quality is in most cases between 1 × 10^−4^/10^−5^ that corresponds to an amount between 2 and 3 logs below the threshold of the achievement of CCyR [13]. As we will see, the achievement of a very low number of leukemic cells is associated with the possibility to try to discontinue the therapy with a substantial probability to remain in remission without the need to restart the therapy [14–16].

Finally, it has been established that mutations in the BCR-ABL kinase domain (KD) represent a frequent phenomenon associated with resistance to TKIs [17]. The early detection and characterization of these mutations may allow timely and appropriate treatment intervention to overcome resistance.

## Cytogenetic and Molecular Milestones to Be Achieved in CML Therapy

**Fig. 1:**
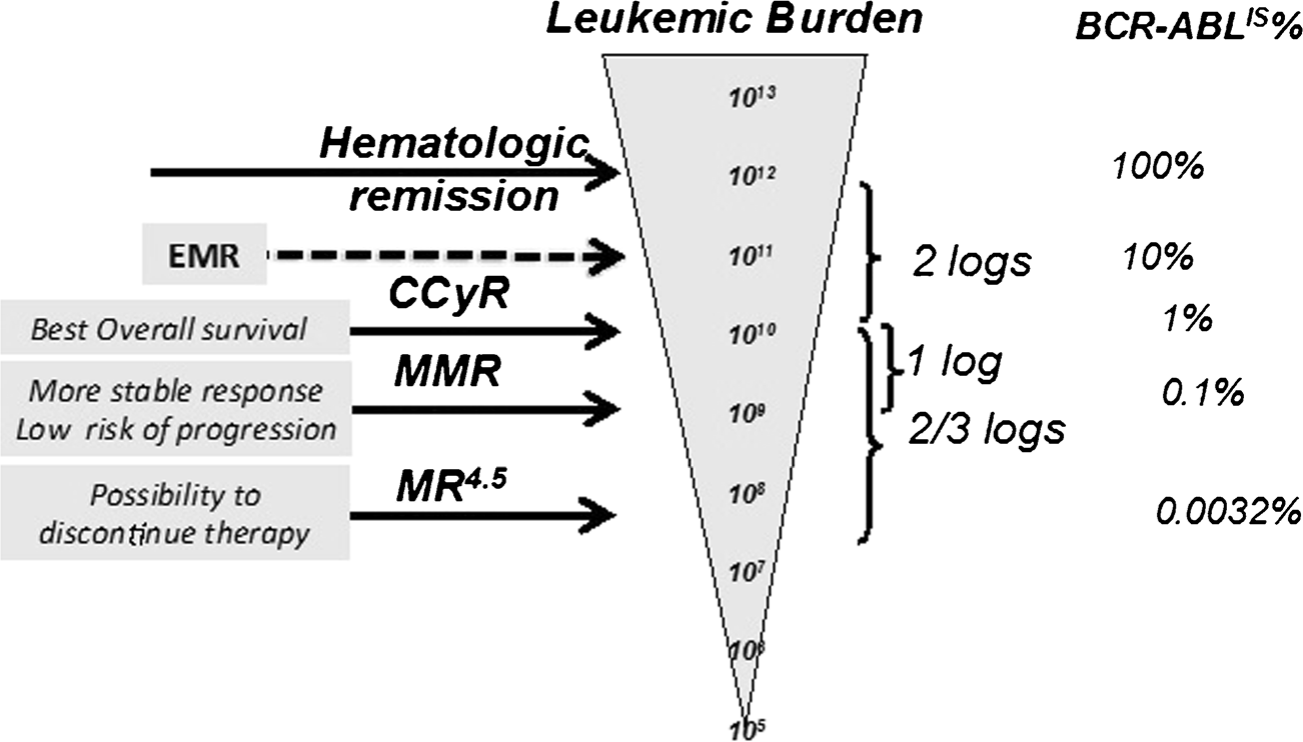
Monitoring of molecular response in CML by RQ PCR. Hierarchic order of responses and corresponding clinical outcomes

In the more recent version of both ELN and NCCN recommendations, to match the “optimal response” definition, the relevant BCR-ABL% to be reached are the following: (a) at 3 months 10 % BCR-ABL according to the established international scale (IS) that represents 1 log reduction with respect to the median BCR-ABL amount present at diagnosis and that roughly corresponds to the threshold of partial cytogenetic response PCyR; (b) at 6 months 1 % BCR-ABL^IS^ that represents 2-log reduction with respect to the median BCR-ABL amount present at diagnosis and that roughly corresponds to the threshold of complete cytogenetic response CCyR); and (c) at 12 months 0.10 % BCR-ABL^IS^ (major molecular response (MMR)), later on to maintain MMR or to reach 0.01 % and to 0.0032 % BCR-ABL corresponding, respectively, to MR^4^ (4-log reduction) and MR^4.5^ (4.5-log reduction) [5••, 6••] (Fig. 1).

From the practical point of view, even in our days probably the attainment of CCyR or 1 % BCR-ABL can still be considered the most significant response to target, as this goal has been demonstrated to be associated to the highest probability of long-term survival for CML patients who better benefit from the TKI therapy are those who achieve and maintain CCyR for at least 2 years, as in these cases the OS is similar to that of a control population without leukemia [7•, 8, 9, 18]. However, whereas in previous editions of the ELN recommendations, this goal was expected at 12 months from start of therapy, in the last versions of both guidelines, CCyR or 1 % BCR-ABL^IS^ is expected already after 6 months of therapy only. This was based on the observation that the landmark analysis at 6 months of the patients in CCyR or not are equally well predictive for PFS and OS than that at 12 months with the advantage that of course the analysis is at an early time point and it is able to predict for more events [7•].

Several sets of data did not appear to support the notion that deeper responses, as the achievement of level of BCR-ABL^IS^ ≤0.1 % (MMR) may indeed improve OS relative to achieve CCyR without MMR [7•, 8]. More recently, however, a 4-year landmark analysis performed within the context of the German CML study IV suggests that the patients who after 4 years were able to achieve a stable MR^4.5^ molecular response, at 8 years show a statistically significant better survival with respect to those patients who have simply achieved CCyR but not MMR [9]. If these results will be confirmed, MR^4.5^ will represent a new molecular predictor of long-term outcome.

In any case, it is has been clearly established by several clinical studies that a stable deep molecular response (at least MR^4^ or even better MR^4.5^) is requested to obtain a long-lasting treatment-free remission (TFR) that is progressively becoming the new treatment goal for many CML patients [14, 15]. Thus, the achievement of MMR^4^ and of MR^4.5^ in addition to CCyR and MMR are appealing targets to pursue, as they predict for more durable and stable responses and can also open the possibility to try to stop the therapy.

It is noteworthy that many studies, particularly in more recent years, have indicated that early cytogenetic and early molecular responses (EMR) within the first year of therapy represent the strongest prognostic parameters [19•, 20–22]. This is not only in terms of OS, progression-free survival (PFS), or event-free survival (EFS) but also in terms of possibility of achieving deeper molecular responses and therefore the possibility of discontinuing treatment without molecular relapse (TFR). Based on these observations, the last editions of the ELN and NCCN recommendations have been modified with respect to the past, the time points at which the expected response goals should be met to match the criteria for optimal response [5••, 6••]. Whereas previously only hematologic remission and some degree of cytogenetic response were expected after 3 months of TKI therapy, partial cytogenetic response (PCyR) after 6 months and CCyR after 1 year, in the last editions of both ELN and NCCN recommendations, to be considered “optimal responders,” the patients should at least be in partial cytogenetic response (PCyR) and/or below the roughly corresponding 10 %^IS^ BCR-ABL threshold after 3 months of therapy, at least in CCyR and/or below the 1 %^IS^ BCR-ABL level after 6 months of therapy and at least in MMR after 1 year of therapy and thereafter show a continuous decline of the BCR-ABL level until the achievement of deeper responses like MR^4^ or MR^4,5^ [5••, 6••].

Indeed, many studies suggest that the most clinically relevant target to be achieved during TKI therapy is represented by a reduction of the BCR-ABL transcript level below 10 %^IS^ at 3 months, as this is associated with a high statistically significant difference in terms of OS and PFS [19•, 20–22].

## Parameters to Change the TKI Therapy

The reasons underlying the decision of changing TKI therapy may be different. In addition to the cases of overt failure and therefore at higher risk of progression and of death, in general 10–12 % of patients may show adverse events (AEs) and become intolerant to treatment with a given TKI and should be moved to the treatment with another drug [23]. Considering all together the reasons leading to discontinuation of a specific TKI, several reports have shown that after 8 years from diagnosis, only approximately 55–60 % of the patients who started with imatinib are still on treatment with this drug [24]. Not a substantial difference has been observed among patients who started therapy with second-generation TKIs as first-line therapy [25, 26]. Therefore, the present-day possibility to have at disposition several TKIs with different characteristics and different toxicity profiles to be used as first-, second-, or third-line therapy represents a great advantage with respect to the past.

However, the most difficult decision to be taken is when to change therapy in case of “nonoptimal response.” Based on the parameter of a cutoff of 10 %^IS^ BCR-ABL at 3 months, it appears that approximately one third of CML patients do not show an optimal response to imatinib therapy and they are therefore facing a statistically significant higher risk of an inferior outcome in terms of EFS, PFS, and also OS (approximately 80 % at 5 years with respect to >95 % of those below 10 % BCR-ABL at 3 months) [19•, 20–22]. This percentage is much lower (approximately 10–15 %) among the patients who started first-line therapy with the second-generation TKIs, but the outcome of these patients is probably even worse with respect to those who do not obtain the response with imatinib [21, 22].

On this aspect, there are discordant indications in the last version of the ELN with respect to what was recommended in the last version of the NCCN guidelines [5••, 6••]. Whereas the latter, for the cases who do not achieve a 10 %^IS^ BCR-ABL cutoff at 3 months, suggests to change TKI therapy, in the ELN recommendations, a more delaying position is suggested for those that are simply above 10 % but not yet overt failures. From the practical point of view, the ELN recommendations suggest simply to look more carefully at those cases by increasing the frequency of the RQ PCR tests and to change therapy only if at 6 months the percentage of BCR-ABL is above 10 %, at this time considered failure [5••].

Actually, it is true that most of these patients (approximately 80 % of those first-line treated with imatinib) will only show a delayed response and that, in case of an overt failure, they will simply require a switch to treatment with a second-generation TKI to achieve a good response in at least 40–50 % of the cases [27, 28]. However, it should also be considered that approximately 15–20 % of them in a short time will progress to a more advanced phase of the disease and will die [19•, 20–22]. Most of these progressions occur in patients who at diagnosis were classified as high or intermediate Sokal’s risk group and progressions are rare in the low Sokal’s risk group in TKI-treated patients. This may be related to the observation the percentage of the patients who do not show an optimal response to imatinib may vary according to the initial clinical and hematological features that determine their initial risk category as established by the Sokal’s, Euro, and also by the more recent EUTOS score [29–31]. In the IRIS study, patients with low-, intermediate-, or high-risk Sokal’s score showed significantly different response rates [32]. In the ENESTnd study, a trial comparing imatinib versus nilotinib as first-line treatment for CML, shows that the use of more potent TKIs as those of second generation can indeed greatly increase the percentage of the patients who at 3 months achieve a value of BCR-ABL trascripts below the 10 %^IS^ in the high and intermediate Sokal’s risk groups: the percentage of the high-risk group patients achieving a value of BCR-ABL^IS^ <10 % when treated with nilotinib (85 %) is almost double with respect to what is observed in the group treated with imatinib (43 %) [33]. This may explain the reduction of the progressions within the first months after start of therapy observed in the patients treated with second-generation TKIs with respect to those treated with imatinib that is particularly evident in the intermediate and high-risk Sokal’s groups, and this also represents the rationale of several clinical trials aiming to improve the first-line treatment of CML patients in order to decrease the number of the patients not achieving an optimal response [34]. The therapeutic strategies so far tested include first-line administration of the second-generation TKIs originally used as second-line therapy or modified imatinib-based regimens, as higher dosages of imatinib from the start or combinations of imatinib with other drugs, namely interferon-alpha (IFN-α) [34]. At present, only the use of the second-generation TKIs nilotinib at the dosage of 300 mg BID and dasatinib 100 mg OD has been approved and registered as first-line therapy in several countries and is also included in the ELN and NCCN recommendations, whereas the other options still remain investigational [5••].

More recently, it has been suggested that evaluation of the so-called halving time at 90 days of therapy (i.e., at least a halving of the BCR-ABL percentage at 3 months with respect to that observed at diagnosis) may represent a useful way to discriminate among imatinib-treated patients those patients who are at real risk of failure to the imatinib therapy and should therefore change TKI therapy, from those who are simply late responders and can therefore remain on the same therapy [35]. Similar data have been reported by RQ PCR analysis using as control gene GUS instead of ABL, in order to have a more exact evaluation of the real amount of the disease burden present at diagnosis [36]. In this case, however, the best discriminating cutoff at 3 months is to reach a value of approximately one third with respect to that present at diagnosis.

It is relevant however that, independently from the risk of progression and of death, those that at 3 months show values of BCR-ABL above 10 % have very scanty possibility to achieve later on within a reasonable lapse of time a deep molecular response (MR^4^ and MR^4.5^) that are conditions necessary to have chances to remain in TFR after TKI therapy discontinuation. This is true also for those patients who have a good halving time but that remain above 10 % after 3 months of therapy [35]. Indeed, to obtain a high rate of deep molecular responses, BCR-ABL^IS^ should be already ≤1 % at 3 months, as also those patients who at 3 months are between 10 and 1 % of BCR-ABL^IS^ have lower possibilities of achieving MR^4^ or MR^4.5^ in a reasonable period of time [21, 22].

In summary, present CML treatment guidelines include EMR at 3 months as the first landmark for evaluating responses to TKI therapy, as BCR-ABL^IS^ levels at this time are key predictors of long-term outcomes for CML patients not only in terms of PFS and of OS but also in terms of the possibility of achieving later on the deep molecular responses that have been associated with additional long-term benefits, including the possibility of the suspension of the TKI therapy. Certainly, the 3 months BCR-ABL^IS^ level thresholds associated not only with an improved PFS and OS but also with an increased possibility of achieving MR^4^ and MR^4.5^ are more frequently obtainable with the use of second-generation TKIs with respect to imatinib and we still do not know whether a change in therapy at this time can really improve the final outcomes of the patients. This, on the contrary to the NCCN guidelines, is the main reason for sustaining the ELN recommendation of a delayed decision to change therapy in absence of an overt failure. However, a flexible position is probably advisable. The change of TKI therapy should be decided in the individual cases considering the goal expected to be achieved, the probability of achieving that goal in a given patient, and the final balance between the possible advantages and disadvantages, including the risk of toxicity and the economic cost, that the achievement of the goal may require.

## Detection of BCR-ABL Kinase Domain Mutations

The presence of BCR-ABL1 kinase domain point mutations is suggestive of genetic instability and of increased risk of progression and is detectable in about 50 % of patients with treatment failure and progression [17]. More than 80 amino acid substitutions have been reported in association with resistance to imatinib. Nilotinib-resistant patients were most frequently found to have acquired Y253H, E255K/V, F359V/C/T315I mutations, whereas dasatinib-resistant patients were found to have acquired V299L, F317L/V/I/C, T315A, and T315I mutations. The spectrum of mutations conferring resistance to bosutinib is similar to that of dasatinib. Therefore, no second-generation TKIs are capable to inhibit the T315I mutation that is only inhibited by ponatinib [37].

Mutational analysis should be performed in all cases of treatment failure, of progression to accelerated phase or blast crisis, in all the cases in which a consistent and confirmed increase of the BCR-ABL transcript level is observed, and in all cases in which a change of TKI therapy is performed [5••, 6••]. The mutation analysis in case of warning is not recommended at the moment, as no data are available to provide evidence that the analysis may be clinically significant in this situation, but of course, it can be performed in specific cases according to the clinicians’ discretion.

Finally, to date, the presence of mutated clones should be assessed using low-sensitivity techniques (Sanger sequencing) [38]. In our days, the presence of very small subclones with mutations can be identified with more sensitive techniques, such as mass spectrometry or ultra-deep sequencing, but data are not yet sufficient to interpret the clinical relevance of the mutations detected by these more sensitive techniques.

## Considerations and Conclusions

CML treatment is undergoing a profound evolution during the last years. This is due to the fact that, besides imatinib, more TKIs of second and of third generation with different efficacy and toxicity profiles are now available and that the final endpoint of the therapy in a rather consistent percentage of patients can be represented not only by the best possible overall survival with respect to a control population without leukemia but also by achieving this goal without the need to assume TKIs for the rest of the life. Because of this, not only the choice of first-line treatment of CML in chronic phase but also the way to harmonize the sequential use of the different TKIs at disposition are at the moment the most hot topics of debate among hematologists dealing with CML around the world.

The present recommendations indeed are bound to highlight and to recommend only elements that have been sufficiently proven in the literature, but of course, they cannot cover all the different situations that are faced by clinicians in real life. That is why a careful observation of the profile of the individual patient in order to decide the therapy and its possible modifications during follow-up is becoming the most important and stringent recommendation. Of course, decisions should be taken also on the basis of a precise evaluation of the disease status and burden, both at diagnosis as well as in response to treatment. On this purpose, precise and standardized methods are needed and at the moment RQ PCR analysis, even more than cytogenetic analysis, is becoming the method of choice. As a fast initial response may be highly predictive of the patients’ final outcome, a more intense schedule for monitoring the response with cytogenetic and/or molecular analysis within the first semester of therapy is advisable even in common clinical practice, as clearly stated in the ELN and NCCN recommendations [5••, 6••]. The RQ PCR technique is also the only one able to evaluate the achievement of the very low levels of residual disease which are needed to open the possibility of achieving a TFR status [13]. In parallel with the development of new and more potent TKIs and/or of suitable combination therapies that could allow a higher percentage of patients to achieve a TFR status, also a simplification and an automation of standardized methods to assess the residual disease are needed to expand the success of the CML therapy on patients all over the world.
